# Unsupervised Spatial Event Detection in Targeted Domains with Applications to Civil Unrest Modeling

**DOI:** 10.1371/journal.pone.0110206

**Published:** 2014-10-28

**Authors:** Liang Zhao, Feng Chen, Jing Dai, Ting Hua, Chang-Tien Lu, Naren Ramakrishnan

**Affiliations:** 1 Department of Computer Science, Virginia Tech, Falls Church, Virginia, United States of America; 2 Department of Computer Science, University at Albany-SUNY, Albany, New York, United States of America; 3 Google, New York City, New York, United States of America; University of Namur, Belgium

## Abstract

Twitter has become a popular data source as a surrogate for monitoring and detecting events. Targeted domains such as crime, election, and social unrest require the creation of algorithms capable of detecting events pertinent to these domains. Due to the unstructured language, short-length messages, dynamics, and heterogeneity typical of Twitter data streams, it is technically difficult and labor-intensive to develop and maintain supervised learning systems. We present a novel unsupervised approach for detecting spatial events in targeted domains and illustrate this approach using one specific domain, viz. civil unrest modeling. Given a targeted domain, we propose a dynamic query expansion algorithm to iteratively expand domain-related terms, and generate a tweet homogeneous graph. An anomaly identification method is utilized to detect spatial events over this graph by jointly maximizing local modularity and spatial scan statistics. Extensive experiments conducted in 10 Latin American countries demonstrate the effectiveness of the proposed approach.

## Introduction

**Figure 1:**
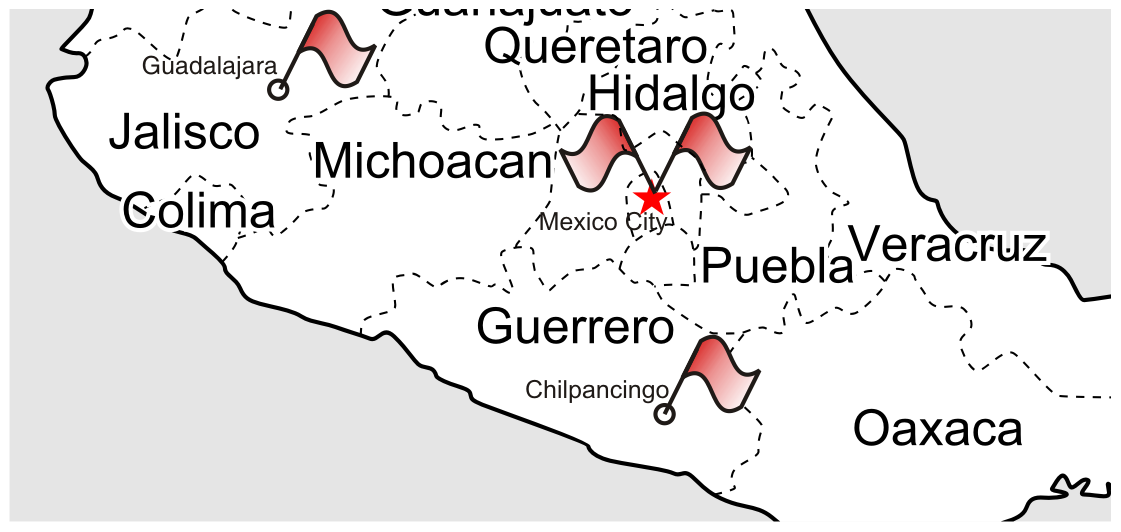
A map of civil unrest event hotspots on September 27th, 2012 pertaining to labor reform and other issues. Flags denote the ground-truth events reported by authorities. Circles denote the events detected by our method.

Microblogs such as Twitter and Weibo are experiencing an explosive level of growth. Millions of worldwide microblog users broadcast their daily observations on an enormous variety of domains, e.g., crime, sports, and politics. Traditional media, in contrast, is monopolized by closed groups, and on occasion may even be under threat from criminal organizations in localities suffering from conflicts and high crime rates [1]. When a social event occurs, it usually takes hours or even days to be reported by traditional media, which is why social media like Twitter have come to play a major role as a real-time information platform for social events [2], [3]. Beyond items of public interest, event-related microblogs can provide highly detailed and timely information for those interested in public safety, homeland security, and financial stability. Figure 1 depicts event hotspots related to the protests on September 27th, 2012 in Mexico. Based on tweets posted on that day, the new approach proposed here automatically and immediately identified these events, some of which were not reported by traditional media until several days later.

Although identifying events from news reports has been well studied [4], analyzing tweets to reveal event information requires more sophisticated techniques. Tweets are written in unstructured language and often contain typos, non-standard acronyms, and spam. In addition to the textual content, Twitter data forms a heterogeneous information network where users, tweets, and hashtags have mutual relationships. These features of Twitter data pose a challenge for event detection methods developed for traditional media. Although there has been a considerable body of work on event detection in Twitter, most of the work published has targeted events of *general interest*. Methods for *general interest* events typically focus on the “hotness” of events but are not sufficient for tracking events in specific domains. It is of high social significance to continuously and closely monitor crucial domains such as crime [5], earthquakes [6], civil unrest [7], and disease outbreaks [8]. Existing methods in event detection suffer from the following shortcomings: 1) their restricted ability to model heterogeneity and network properties of Twitter data. Existing methods typically treat Twitter data as a set of plain textual documents. However, “tweet”, “word”, “hashtag”, and “user” are of different entity types. For example, a “user” can post a “tweet”, “tweets” can be tagged by a “hashtag” and a “tweet” can reply to another “tweet”. In general, these heterogeneous relationships and properties are not effectively harnessed by existing methods; 2) their limited ability to handle the dynamic properties of Twitter data. Existing methods treat fixed keywords as features for classifying tweets. However, the expression in tweets dynamically evolves, which makes the use of fixed features and historical training sets inappropriate. For example, the most significant Twitter keyword for the Mexican protests in Aug 2012 was “#YoSoy132” (i.e., the hashtag of an organization protesting against electoral fraud), alluding to the protests against the Mexican presidential election, but “#CNTE” (i.e., the hashtag stands for the national teacher's association of Mexico) had become the most popular term by the beginning of 2013 due to the movements against the Mexican education reform; and 3) their inability to jointly model the semantic similarities and geographical proximities of events. Existing methods generally cannot differentiate between multiple events that occur simultaneously in the same location. For instance, in Mexico City, from Jan 30th, 2011 to Dec 31th, 2012, there were a total of 116 civil unrest events on 83 dates, of which 25 dates involved multiple events. On Sep 27, 2012, two different protests occurred in Mexico City, organized separately by “#Yosoy132” and “City sanitation workers”. Hence, without the capacity to distinguish events' semantic contexts, existing methods typically miss nearly 30% of the events occurring in Mexico City.

In this paper, to address the above-mentioned issues, we present an unsupervised “targeted domain” spatial event detection method that can jointly handle the heterogeneity and dynamics of Twitter data. Our contributions are summarized as follows:

- **Development of an unsupervised framework:** We propose a novel unsupervised approach for targeted domain spatial event detection in Twitter. Our method requires no intensive human labor such as training set labeling.
- **Design of a novel dynamic query expansion (DQE) method:** Given a targeted domain, DQE dynamically generates a set of domain-related key terms via a Twitter heterogeneous information network. The key terms are exhaustively extracted and then weighted appropriately based on DQE's iterative process.
- **An innovative local modularity spatial scan (LMSS) algorithm.** Based on a graph formed using key terms from DQE, LMSS jointly maximizes the local modularity and spatial scan statistics in order to distinguish events by taking into account both their semantic similarities and geographical proximities.
- **Extensive experimental evaluation and performance analysis.** Our method was extensively evaluated on Twitter data covering 10 Latin American countries. Comparisons with baselines and state-of-the-art methods demonstrated its effectiveness and efficiency.

## Materials and Methods

### Literature Review

Current microblog-based event detection methods can be classified into two categories: 1) *general-interest event detection*, and 2) *targeted-domain event detection*.

#### General-interest event detection

Methods under this category aim to detect emerging general-interest topics in the Twitter data stream, and typically apply unsupervised techniques such as topic modeling, burst detection, and clustering techniques. Yin et al. [9] developed geographic topic modeling techniques to detect topics clustered in local geographic regions, while Lappas et al. [10] proposed methods to discover bursts of terms in a specific spatial and temporal neighborhood. Weng et al. [11] applied wavelet analysis for noise filtering and then identified word groups with high correlations, each of which is returned as the indicator of an event. Adopting a different approach, Aggarwal and Subbian [12] developed an algorithm that captures the related signals by considering the tweets' content, network structural, and temporal information. Finally, Ritter et al. [13] suggested an NLP-based approach to general event extraction from twitter data.

#### Targeted-domain event detection

Methods under this category aim to detect events within a particular field, e.g., “earthquakes”, “disease outbreaks”, and “civil unrest”. These methods generally rely on supervised learning techniques like the support vector machine (SVM). Human labor is required to label the subsets of tweets related to the targeted domain, and then clustering techniques are applied to identify the locations of the events. An example of this is a study by Sakaki et al. [6], who designed a classifier to extract earthquake-related tweets and then utilized Kalman filtering to detect the geographic regions where the earthquakes had occurred. For tracking disease activities, Signorini et al. [8] adopted an SVM classifier to extract tweets related to various types of disease, while Chakrabarti et al. [14] trained a modified Hidden Markov Model to learn the structure and vocabulary of sports-related tweets, which were then utilized to generate summaries of the sports events. Li et al. [5] trained a classifier to extract crime-related tweets, first sorting the tweets based on their importance, and then applying them to detect crime events.

### Problem Formulation

Twitter data contains heterogeneous entities and multiple types of relationships, which can be formulated as a Twitter heterogeneous information network:

**Definition 1** ***(Twitter Heterogeneous Information Network)*** *A* **Twitter heterogeneous information network** *is defined as an *undirected *graph* 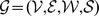, *where* 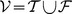. 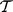 *refers to a set of* **tweet nodes**, *and* 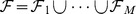 *refers to other* 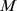 *types (e.g., term, user, and hashtag) of nodes, called* **feature nodes**. 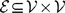 *represents the set of edges, which are all undirected. We denote the existence of an edge between two nodes* 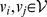 *by* 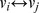. 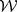 *denotes the set of weights of nodes and edges*. 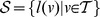 *refers to a set of geographic locations of tweet nodes, where* 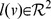 *represents a tuple consisting of the latitude and longitude of tweet node v*. *When M = 0,* 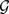 *reduces to a* **Twitter homogeneous information network** 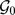.

In addition to ***tweet nodes***, we consider several other types of nodes, including “term”, “hashtag”, “hyperlink”, and “user”, all of which are generally called ***feature nodes***. The relationships between these types of nodes are denoted by the set of undirected edges 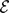, including *authorship* between user nodes and tweet nodes, *containment* between tweet nodes and term nodes, and *replying* between tweet nodes.

**Definition 2** ***(Seed Query)*** *A* **seed query** *is defined as an initial set of semantically coherent feature nodes that characterize the concept of the targeted domain. A seed query is denoted as* 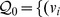, 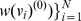, *where the feature node v_i_ is a* **seed query term** *whose weight* 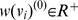 *reflects its relevance to the targeted domain. An* ***expanded query*** *is an extended set of weighted feature nodes that represent the semantic contexts of spatial events. Similar to seed query, an expanded query is denoted as* 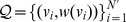, *where v_i_ is called an* **expanded query term**.

All the *seed query terms* have corresponding edges denoting their semantic relevance. For example, given a seed query of the domain “civil unrest”: {(“protest”, 1), (“march”, 1), (“strike”, 1), (“unrest”, 1)}, an expanded query can be: {(“#megamarcha”, 0.1), (“#YoSoy132”, 0.3), (“zocal”,0.1), (“march”,0.2), (“imposición”,0.1)}, which matches the news description: “A mega march against the imposition of PRI: YoSoy132 protestors arrived at El Zocalo.”

Denote 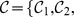 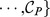 as a collection of time-indexed Twitter data, where 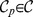 represents the subcollection of tweets posted between timestamps 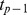 and 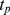. To achieve targeted domain spatial event detection, one needs to concentrate on domain-related tweets and detect the spatial burst signals based on them. The major tasks of the **targeted-domain spatial event detection** problem are defined as follows:

**Task 1: Expanded Query Generation:** Given 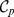 and a seed query 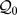 of a targeted domain, ***expanded query generation*** is to generate the expanded query 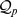 by expanding 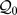 through the Twitter heterogeneous information network 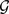.

**Task 2: Spatial Event Extraction:** Given a targeted-domain related tweets subset extracted based on 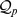 from 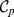, ***spatial events extraction*** is to automatically identify a set of spatial events, each of which is specified by geolocation, time, and related tweet nodes.

### Dynamic Query Expansion

**Figure 2:**
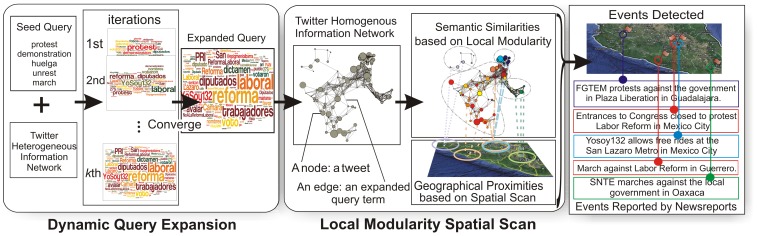
Flowchart of the proposed method.

Specially designed for Twitter data, the dynamic query expansion (DQE) algorithm utilizes heterogeneous relationships (e.g., containment, authorship, and replying) extracted from the Twitter heterogeneous information network to expand the seed query. The leftmost component in Figure 2 shows the general framework of the DQE algorithm.

#### Calculation of Relevances to Targeted Domain

Given a seed query, we must first focus on generating an expanded query. Traditional query expansion methods generally expand the seed query by examining the terms' semantic or co-occurrence relationships in textual documents [5], [15], [16]. To further enhance the coverage of expanded query, recently Li et. al proposed to expand the query iteratively by taking into account the usefulness and coverage of the keywords in each iteration [17]. However, their method is not guaranteed to converge and is sensitive to the number of iterations user specified. Most existing query expansion methods are seriously challenged by the heterogeneity of Twitter data. *First, Twitter data contains multiple types of entities.* In addition to terms, entities such as “users”, “hashtags”, and “hyperlinks” are all important for revealing the implicit relevance between tweets. For example, a “keyplayer” (i.e., an important Twitter user in a particular domain of activity) in a particular domain will frequently post domain-related tweets, and thus the tweets and terms posted by him/her are likely to be domain-related. *Second, Twitter data contains heterogeneous relationships among multiple types of entities.* Social relationships in Twitter provide heuristics to associate tweets under a same domain. For example, tweets can have mutual social relationships such as “replying” or “replied”. A tweet and the tweets replying to it will therefore generally fall into the same domain.

To overcome these challenges, we propose a new dynamic query expansion algorithm that utilizes the heterogeneous relationships of Twitter heterogeneous information network. Referring to Definition 1, for any node 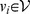, its weight 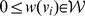 is defined as its relevance to the targeted domain. The nodes with higher weights are more relevant to the targeted domain. For example, “protest” and “#OccupyWallSt” are more relevant to the “civil unrest” domain than “love” and “#music”, thus the weights of “protest” and “#OccupyWallSt” are higher. To simplify the notation, for any 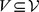, the weights set 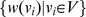 is denoted as 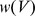.

Heterogenous relationships among entities are ubiquitous and important in Twitter. Terms such as “protest” are deemed to be related to the “civil unrest” domain because they appear frequently in the set of domain-related tweets. Similarly, user “ESPN” is related to the “sports” domain because “ESPN” mainly posts tweets about sports; tweets tagged by the hashtag “#OccupyWallSt” are considered to be about “civil unrest”. A tweet is typically deemed to be in the same domain as the one it replies to. Tweet nodes and feature nodes generally exhibit a mutual reinforcing relationship. Given a set of feature nodes 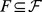 and tweet nodes 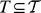, if a feature node 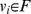 has edges with many high-weight tweet nodes instead of low-weight ones, it should receive a large weight value. Then, if 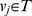 has edges with many high-weight feature nodes, it should also be assigned a large weight value. It also follows that, if 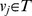 has a replying relationship with a high-weight tweet node 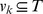, 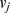 should also receive a large weight value. The first of these relationships determines the weights of feature node set *F* while the second and third determine the weights of tweet node set *T*.

The operation to determine the weights of the nodes in *F* proceeds as follows:
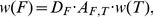
(1)where 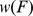 and 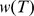 denote the vector weights of *F* and *T*, respectively. 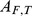 denotes the adjacency matrix between *F* and *T* such that 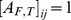 if 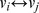, where 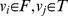; 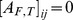, otherwise. 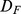 is the inverse document frequency (IDF) [11] matrix of *F*, which is a diagonal matrix such that 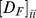 refers to the IDF of 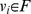.

The operation to determine the weights of the nodes in 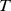 proceeds as follows:
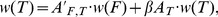
(2)where *β* reflects the tradeoff between the influences of feature nodes and tweet nodes on the calculation of 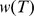. 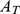 denotes the matrix of the replying relationship between tweet nodes such that 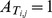 if 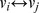, where 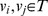; 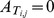, otherwise. 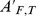 is the transpose of matrix 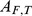.

#### DQE Algorithm Description

To generate an expanded query, above-mentioned operations are utilized via an iterative DQE algorithm, as shown in Table 1 Algorithm 1. The major issues of the algorithm implementation are described in the following.

**Table 1:** The algorithm of Dynamic Query Expansion.

Algorithm 1: Dynamic Query Expansion.
**Input**: Seed Query 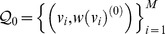 , Twitter subcollection 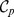
**Output**: Expanded Query 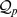
Initialize *T*, *F*, 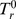 , and 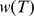
Set Φ via Equation 3 and 4
Set *k* = 0
**repeat**
** repeat**
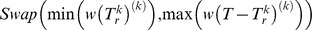
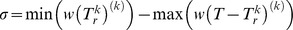
** until** *σ*≥0;
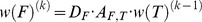
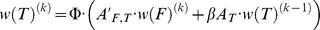
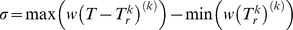
* k* = *k*+1
**until** *σ*≤0;
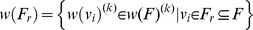
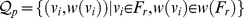 .

***Initialization*** **.** Suppose we are given a seed query 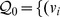, 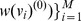 for the targeted domain. Denote 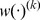 as the weight(s) of the node(s) at the *k*th iteration. Denote 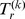 as the set of domain-related tweet nodes at the *k*th iteration. To trigger the iterative operations, 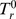 is initialized as the set of tweet nodes matching 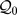. All the feature nodes having edges with nodes in 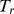 are potentially domain-related and thus can be used to initialize the feature node set 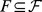. The initial tweet node set 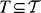 consists of tweet nodes, each of which has edge(s) with at least one node in *F*. Naturally, 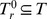. 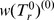 is an all-one vector while 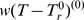 is a zero vector.

***Stopping Criterion*** **.** For the *k*th iteration, tweet nodes in 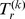 are compared to those in 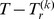 based on their weights. If 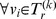 and 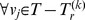 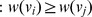, then the iterations will be terminated, as shown in Line 13. Otherwise, the lowest-weight node in 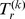 will be exchanged with the highest-weight node in 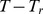 (denoted by the function “Swap” in Line 6) until 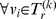 and 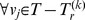 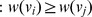, as shown in Line 8.

***Generation of the Expanded Query*** **.** After the iterations are completed, the ultimate set of domain-related tweet nodes is 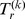. Define a set 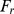 of feature nodes, each of which has edge(s) to at least one node of 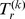. Due to 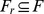, the weights of the nodes in 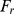 have been calculated, as shown in Line 14, and eventually the expanded query 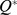 is formed in Line 15.

***Analysis of Convergence*** **.** Equations 1 and 2 are combined to capture the weight updating of *T*:
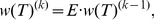
(3)where the matrix *E* is a transition matrix (column-normalized by 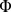) consisting of the relevances between any two tweet nodes in *T*:
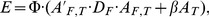
(4)where 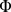 normalizes 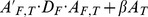 by column so that the weights in 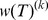 sum to a constant.

Formulate three facts introduced above: 1) 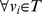, 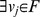: 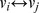, 2) 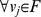, 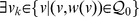: 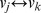, and 3) 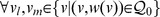: 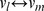. Therefore, we obtain 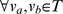, 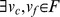 and 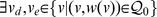 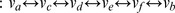, which means any two nodes in *T* have a path connected to each other. Hence, *E* is irreducible because its corresponding graph formed by *T* is strongly connected [18].

The Markov chain associated with *E* is irreducible. In addition, its aperiodicity is guaranteed [19]. Therefore, this Markov chain is ergodic. Based on the stability theorem of Markov chains, the existence of a unique stationary distribution vector for this Markov chain is guaranteed [18], which means as *k* increases, 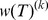 converges to 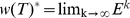. Therefore, the convergence is guaranteed.

### Local Modularity Spatial Scan

We describe a local modularity spatial scan (LMSS) model that can be applied to extract spatial events, as illustrated in the corresponding component in Figure 2. Based on the tweet graph built with the expanded query, we first derive an optimization function for identifying anomalous subgraphs, and then apply this function to identify tweets related to latent spatial events.

#### Anomalous Subgraph Identification

The expanded query 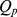 contains the feature nodes that are most relevant to the targeted domain. 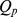 is utilized to retrieve the set of domain-related tweets 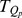, in which each tweet contains at least one of the expanded query terms. We need to extract tweet node sets 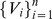, where each 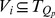 contains tweets related to a latent spatial event. This is typically solved by spatial clustering methods [5], [6]. However, if only the geographic proximities in clustering are considered, it is not possible to distinguish between discrete events when they occur in the same location.

To address this problem, the semantic similarities and geographical proximities of tweets are jointly considered based on the Twitter homogeneous information network. The event-related tweets need to be both semantically similar and geographically close. Specifically, by referring to Definition 1, we can build a Twitter homogeneous information network 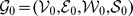, where 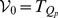 denotes the node set, 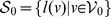 stands for the tweet nodes' geographic locations, and 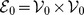 represents the set of undirected edges. In addition, the weight set 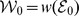 represents the semantic similarities among tweet nodes such that two tweets are semantically similar if they share expanded query terms. Mathematically, 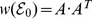 where *A* is the adjacency matrix between 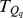 and 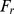. Since 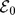, 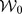, and 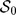 all depend on 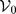, for convenience we denote 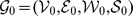 as 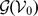. The graph 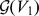 is said to be a subgraph of graph 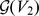 if 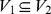. Hence, in 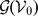, the event-related tweets are deemed to compose a subgraph 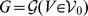 that satisfies two properties: 1) tweets in *G* connect via high-weight edges, and 2) tweets in *G* are geographically proximate with each other.

For the first property, local modularity [20] is adopted, which is a metric generally applied to measure the quality of a connected subgraph:
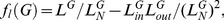
(5)where 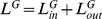. 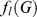 is the local modularity of 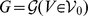, an arbitrary subgraph of 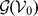, 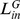 refers to the sum of the weights of the edges in *G*, 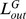 denotes the sum of the weights of the edges that connect nodes in *G* and nodes outside *G*, and 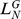 represents the sum of the weights of the edges in the subgraph formed by the nodes in the geographical neighborhood of the nodes of *V*.

For the second property, Kulldorff proposes an effective metric to measure the geographical proximities of a spatial cluster, dubbed the Kulldorff statistic [21]. It is applied to measure the geographical proximities of the subgraph *G*:
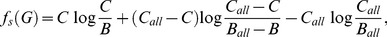
(6)where *C* refers to the count of tweet nodes in 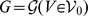, *B* refers to the size of the set of tweet nodes 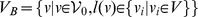, 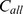 denotes the count of tweet nodes in 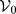, and 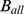 represents the count of tweet nodes in 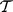.

Hence, Task 2 can be addressed by identifying the anomalous subgraphs that jointly maximize the preceding two quality metrics. This is formalized as a multi-objective optimization problem as follows:
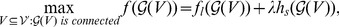
(7)where 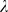 is a predefined parameter to balance the significance of the local modularity for semantic similarities and the Kulldorff statistics for spatial proximities. 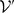 is an arbitrary subset of 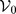.

#### LMSS Algorithm

By exploring the linear-time subset scanning (LTSS) property of the Kulldorff statistic [22], we propose a fast approximate algorithm (Algorithm 2 in Table 2) that adopts a heuristic strategy to search for anomalous subgraphs that maximize 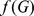 in Equation 7. The algorithm is elaborated as follows.

**Table 2:** The algorithm of Local Modularity Spatial Scan.

Algorithm 2: Local Modularity Spatial Scan.
**Input**: 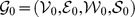
**Output**: 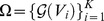 , where 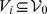
Initialize 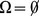
**for** 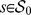 **do**
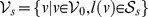
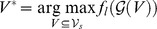 , s.t. 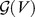 is connected
** repeat**
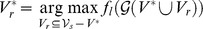 , s.t. 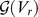 is connected
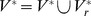
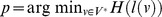
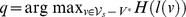
**if** *H*(*p*) <*H*(*q*) **then**
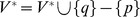
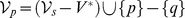
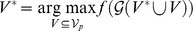 , s.t. 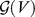 is connected
** until** *H*(*p*) ≥ *H*(*q*);
Add 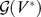 to Ω
Check overlapping among subgraphs and update Ω
Randomization testing on subgraphs and update Ω

***Anomalous Subgraphs Extraction (Line 2–4).*** Each distinct geographic location 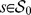 is considered as a candidate geographic center (Line 2). A tweet node set 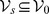 is first extracted with a corresponding set 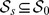 consisting of locations within a distance 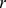 of the center 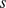 (Line 3). In each 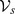, by applying a local modularity graph clustering algorithm [20], a subset 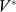 is found that has the maximum local modularity (Line 4).

***Subgraph Refinement (Line 5–15).*** We next find a connected subgraph 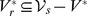 that maximizes 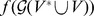 (Line 6), where 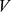 is a subset of 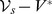. Then 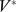 is updated by merging it with 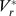 (Line 7). To achieve linear time subgraph scanning, Neill proposed a statistic priority function 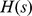 for location 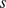 such that 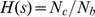, where 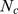 and 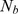 are the numbers of tweet nodes on location 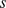 in the subgraph and in the whole graph, respectively [22]. If the minimum value of the statistical priorities of the locations of 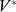 is larger than the maximum value of those of 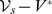 (Line 8–10), add 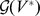 into the graph list 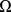 (Line 15). Otherwise, exchange the minimum-value location of 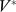 with the maximum-value location of 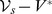 (Line 11–12), and update 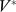 by finding a subgraph 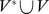 that maximizes 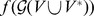, where 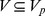 (Line 13).

***Candidate Subgraph Set Pruning (Line 16–17).*** If there exist subgraphs 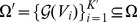 sharing the same nodes, retain only the subgraph 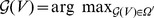 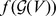 (Line 16). We then test 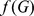 of each subgraph 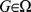 with randomization testing, and retain only the subgraphs with empirical p-values smaller than 0.05 (Line 17).

The LMSS algorithm exhibits several advantageous theoretical properties, as follows:

**Theorem 1** *Algorithm 2 in* *Table 2* *has the following theoretical properties: If* 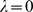 *, it is guaranteed to return a local optimal solution that maximizes the local modularity score* 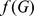 *; If* 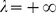 *, it is guaranteed to return a global optimal solution that maximizes the Kulldorff statistic* 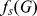.

*Proof.* If 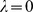, then the solution of Line 4 will be returned as the final value of 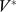 for *s*, which proves the first property in Theorem 1. If 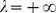, then the Kulldorff statistic dominates 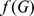. Line 6 searches for the set of tweet nodes 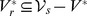 by maximizing 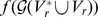. Note that in this step, the set of locations of tweet nodes in 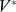 is fixed, and hence the factors *C*, *B* and 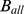 are fixed. Given that 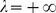 and 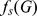 is a homogeneous function of the count *C*, the optimal solution 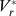 is identical to 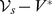. Recall the basic idea of the LTSS property [22]: the subset of geographic locations that maximizes the Kulldorff statistic can be found by ranking the locations according to the priority function 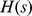, and then searching over groups consisting of 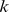 locations with highest priority values. It can be readily proved that by solving the objective function in Line 13, the resulting 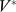 will be the connected subgraph consisting of the locations with the highest priority values. Hence, the Kulldorff statistic 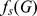 will be maximized. □

### Time Complexity Analysis

The time complexity of DQE is 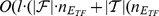 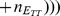, where 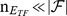 is the average number of connections between a tweet node and feature nodes, 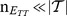 is the average number of connections from a tweet node to other tweet nodes, and 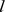 is the number of the iterations of DQE. Typically, 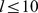.

The time complexity of LMSS is 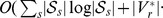 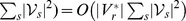, where 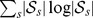 corresponds to the solving of the objective function in Line 6 of Table 2 Algorithm 2 while 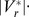 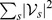 corresponds to the local modularity calculation. 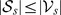 and 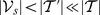, where 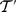 is the set of the tweet nodes with weights higher than 0.

By summing up these two parts, which correspond to DQE and LMSS, respectively, the overall time complexity is 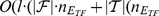 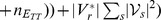.

## Results

In this section, the empirical evaluations of the performance of our approach, DQE + LMSS, are presented. By comparing the results with those obtained using existing methods and baselines, the effectiveness and efficiency of our method and its components are demonstrated. Sensitivity analysis and case studies are also included in this section. All the experiments were conducted on a computer with one 3.20 GHz Intel Xeon CPU and 18.0 GB RAM.

### Dataset and Labels

Twitter data used in this paper was purchased from Datasift Inc. (www.datasift.com). All analyses here are done in compliance with Twitter and Datasift terms of use. The dataset consists of randomly selected 10% tweets of all the tweets sent in the period from July 2012 to May 2013 in the 10 countries listed in Table 3. This dataset was separated into two parts: 1) data from July to October 2012, which served as the training set for the supervised comparison methods, and 2) data from November 2012 to May 2013, which was used as the testing set for validating all the methods. Both the training set and testing set were partitioned into date intervals and event detection was performed for each country individually based on each day's data. Stop-words from tweets were eliminated while stemming was also implemented.

**Table 3:** Dataset and Label Source.

Country	#Tweets (million)	News source^1^	#Events
Argentina	52	Clarín; La Nación; Infobae	365
Brazil	57	O Globo; O Estado de São Paulo; Jornal do Brasil	451
Chile	28	La Tercera; Las Últimas Notícias; El Mercurio	252
Colombia	41	El Espectador; El Tiempo; El Colombiano	298
Ecuador	13	El Universo; El Comercio; Hoy	275
El Salvador	7	El Diáro de Hoy; La Prensa Gráfica; El Mundo	180
Mexico	51	La Jornada; Reforma; Milenio	1217
Paraguay	8	ABC Color; Ultima Hora; La Nacíon	563
Uruguay	3	El Paí; El Observador	124
Venezuela	45	El Universal; El Nacional; Ultimas Notícias	678

1 In addition to the top 3 domestic news outlets, the following news outlets are included: The New York Times; The Guardian;

The Wall Street Journal; The Washington Post; The International Herald Tribune; The Times of London; Infolatam.

Our detection results were validated against a labeled events set, namely the Gold Standard Report (GSR). GSR was exclusively provided by MITRE [23]. The general collection protocol followed by the GSR is as follows: for each country, the top 3 newspapers were selected from among the top 100 newspapers published in Latin America, as provided by International Media and Newspapers. News was also collected from the most influential international news outlets and with additional input from subject matter experts. An event was considered “significant” if it was reported by any of these news outlets. The dataset and labeled news sources for each of these countries are listed in Table 3.

### Methods for Comparison

Table 4 lists all the comparison methods tested: Earthquake Detection, Topic Modeling, Graph Partition, Spatial Temporal Burst (ST Burst), TEDAS, and EDSS. Their implementations and parameters settings were as follows.

**Table 4:** Methods and Efficiencies.

Methods	Targeted Domain	Supervised	Running Time
Earthquake Detection [6]	Yes	Yes	15.2 hours
Topic Modeling [9]	No	No	9.7 hours
Graph Partition [11]	No	No	18.9 hours
ST Burst [10]	No	No	30.1 hours
TEDAS [5]	Yes	Yes	20.9 hours
QE [24] + LMSS	No	No	23.2 hours
SVM + LMSS	Yes	Yes	22.0 hours
DQE + SS [22]	Yes	No	16.3 hours
Our proposed (DQE + LMSS)	Yes	No	18.2 hours
EDSS [12]	No	No	19.8 hours

**Earthquake Detection [6]** **:** This method is initially proposed to detect earthquake, here it is borrowed to detect civil unrest events. 5,386 tweets were manually labeled as “civil unrest related” and another 6,147 tweets as non-related for training purposes. Three types of features were evaluated: statistical, keyword, and word context. All these types of features were tested and the keyword feature were chosen for its best performance.

**Topic Modeling [9]** **:** The implementation was provided by the authors. Hashtags were treated as tags and tweet geotags were deemed to be the corresponding geographic regions.

**Graph Partition [11]** **:** The authors employed a weighted Median Absolute Deviation to handle the skewness of the signal strength distribution. Various weight values from 1 to 40 were evaluated and the value 20 was chosen since it achieved the best performance.

**ST Burst [10]** **:** The implementation was provided by the authors. The tunable temporal window size was set to 6 in the original work. We also evaluated other values, including 12 and 24, but observed similar results.

**TEDAS [5]** **:** The tunable parameters (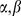) and (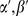) were used to denote the priors to punish words with low frequencies. The well-recognized setting: 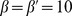 was followed to filter out trivial words. The setting 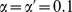 was adopted due to the low percentage of civil unrest content.

**EDSS [12]** **:** The tunable parameter, 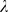, balances the relative significance of content and network structure in event detection. Various values, including the extreme values of 0 and 1 for 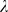, were tested. The setting 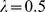 was adopted since it outperformed the other settings tested.

In addition, the effectiveness of each component of our DQE + LMSS was tested by comparing with those of 3 baselines, namely query expansion (QE) + LMSS, support vector machine (SVM) + LMSS, and DQE + spatial scan (SS):

**QE + LMSS:** QE was implemented by following the original design in [24], and adopted the same seed query as that used in DQE.

**SVM + LMSS:** SVM was adopted by following the experimental settings of the “Earthquake Detection” method. Domain-related tweets were extracted based on SVM and then utilized by LMSS.

**DQE + SS:** As a popular spatial scan statistic, the Kulldorff statistic was applied with our DQE to compose a baseline [22].

### Validation

All the comparison methods and baselines returned the event-related tweet content and the corresponding time and location. In addition to the “civil unrest” domain, the general-interest event detection methods output the events under any domains. Therefore, to achieve a fair comparison, events from other domains were filtered out for these methods. In particular, given the “event”-related tweets generated for any method, a linear SVM classifier was adopted to classify all the events into two categories: events in the civil unrest domain and those in other domains. The classifier utilized unigram features, and was trained based on 5,386 tweets manually labeled as “civil unrest related” and another 6,147 tweets labeled as “unrelated”.

After the extraction of “civil unrest” events by the general-interest event detection methods, all the methods were validated against the GSR. A detected event “matches” a GSR event if the following conditions are both satisfied: 1) the event time detected is the same as the time period recorded in GSR; 2) the event location detected is within the same city as that recorded in GSR.

### Initial Settings

There are several tunable parameters in our approach. *β* in Equation 2 is a parameter for updating tweet node weights. Its default value is set to 1. 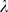 in Equation 7 was used to balance the weights between local modularity and spatial scan statistics, and with 1 as its default value. Other settings of *β* and 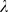 were also studied and are discussed in the rest of the paper.

To initialize DQE, a user is asked to choose 10 civil unrest tweets. In those tweets, terms are ranked based on their document frequency-inverse document frequency (DFIDF) [11] weights. For Spanish speaking countries, the top keywords are: “protesta”, “marcha”, “movimiento”, “patriótica”, “manifiesto”, “violencia”, “holguín”, “americateve”, “cubanet”, and “rolezeiros” in a descending order of DFIDF. Based on our experiments, the top ranked terms are generally related to civil unrest, such as “protesta”, “marcha”, and “movimiento”, whatever the initial 10 civil unrest tweets selected. The same situation applies for Portuguese speaking countries. The top 5 keywords were selected as the seed query terms, all of which were assigned with the same weight to form the seed query. The impact of the number *N* of seed query terms is discussed in the *Study of Parameters* Section.

Additionally, in LMSS, the longest distance *r* between any two neighboring locations was set to 200 km. We also tried 20 additional values of *r* ranging from 150 km to 370 km, and found it made little difference to the performance, as noted in the *Study of Parameters* Section.

### Evaluation of Components

First, the empirical cases will be presented to illustrate the correctness of the expanded query generated by DQE, then the effectiveness of DQE and LMSS are demonstrated based on quantitative comparisons with the baseline methods.

#### Quality Analysis of DQE's Performance

Here, DQE is proposed to generate the expanded queries. Table 5 lists GSR events in July 2012 in Mexico and the corresponding expanded query terms generated by DQE. In the second column, for each date the 6 query terms with the highest weights are listed as the representatives of each expanded query. For each date, the expanded query terms are not only all related to the civil unrest domain, but are also very relevant to the GSR description on that date. Determinative key terms such as event locations, event times, and organization names are successfully identified. Moreover, event-related key hashtags (e.g., “#Megamarcha”) and keyplayers (e.g., “epigmenioibarra”) were also effectively extracted. Interestingly, the only exception was on July 8th, where the key term “Eugenio Derbez”, a popular celebrity in Mexico, was detected. This name became a key term because the protest happened to occur near to his wedding venue, which was reported in online media.

**Table 5:** Comparison between Expanded Query from DQE and GSR Description of Events.

Detect-Date	Expanded Query Extracted by DQE	GSR Description of Real Events	Occur-Date
1-Jul	#YoSoy132, #Granmarcha132, patrull, Companer, PRI, movement	“Youth movement #YoSoy132 staged a sit-in outside the local board of Federal Electoral Institute.”	1-Jul
3-Jul	#Epnnuncaseramipresidente, fraud, #YoSoy132, movimient, progress, contig, march	“The student movement #YoSoy132 protested against fraud in the elections.”	3-Jul
7-Jul	#Megamarcha, #Exigimosdemocracia Eugenio, Derbez, eleccion, @YoSoy132Media	“Protesters unite to call for mega march.” “YoSoy132 go and concentrate on the Esplanade of Heroes.”	7-Jul
8-Jul	#Megamarcha, #Megamarch, Eugenio, Derbez, against, election	“Protesters unite to call for mega march against virtual presidential election.”	
13-Jul	imposicion, #Megamarcha, 15hrs, principal, march, #AMLO	“A march was in protest of the imposition of the PRI candidate.”	14-Jul
14-Jul	#Megamarcha, #Megamarch, 14juli, zocal, angel, march	“Virtual #Megamarch against the winner of the presidential election, Enrique Peña Nieto, left the Angel de Independencia to el Zocalo of Mexico City.”	14-Jul
19-Jul	#Sosmexico, #Sosmexic, fraud, elector, march, protest	“Protesting for alleged fraud in the election of July 1”	19-Jul
22-Jul	#Megamarcha, #YoSoy132, @epigmenioibarra, Zocal, march, imposicion	“A mega march against the alleged imposition of the PRI.”	22-Jul
		“YoSoy132 march arrives at El Zocalo and goes to the Monument to the Revolution”	
27-Jul	#Ocupatelevisa, #YoSoy132, televisa, chapultepec, installation, march	“Students symbolically take over facilities of Hidalgo Radio and TV, and fence outside Televisa Chapultepec in Mexico City”	27-Jul

#### Quantitative Analysis of DQE's Effectiveness

We are interested in examining whether DQE is the best choice for our event detection method, compared to other classic methods. Therefore 2 baseline options, QE and SVM, were introduced as potential replacements for DQE to be used in conjunction with LMSS. The performance of these baselines was then compared with our proposed DQE + LMSS. The results are shown in Table 6. DQE + LMSS achieved the best F-measures in 8 of the 10 countries and was second best in Paraguay and Ecuador. Moreover, it consistently achieved highly competitive F-measures of above 0.5 across all the countries tested, which confirms the stability of its performance. This demonstrates that DQE is a better choice for our event detection method.

**Table 6:** Performance Comparison with Baseline Components (Precision, Recall, F-measure).

Dataset	DQE + LMSS	DQE + SS	QE + LMSS	SVM + LMSS
Brazil	**0.93**, 0.37, 0.53	0.84, **0.59, 0.69**	0.44, 0.14, 0.21	0.39, 0.24, 0.30
Colombia	**0.81, 0.75, 0.78**	0.58, 0.73, 0.65	0.31, 0.16, 0.21	0.63, 0.64, 0.63
Uruguay	0.66, **0.82, 0.73**	0.76, 0.26, 0.39	**0.80**, 0.58, 0.67	0.45, 0.27, 0.34
El Salvador	**0.83, 0.43, 0.56**	0.63, 0.09, 0.16	0.55, 0.37, 0.44	0.61, 0.19, 0.29
Mexico	**0.91, 0.49, 0.6**4	0.73, 0.37, 0.49	0.56, 0.09, 0.16	0.56, 0.18, 0.27
Chile	**0.80**, 0.69, **0.74**	0.58, **0.75**, 0.65	0.28, 0.28, 0.28	0.78, 0.29, 0.42
Paraguay	**0.98**, 0.35, 0.52	0.96, 0.17, 0.29	0.88, **0.67**, **0.76**	0.57, 0.11, 0.19
Argentina	0.78, 0.61, 0.69	0.69, **0.71**, **0.70**	0.67, 0.54, 0.60	**0.92**, 0.22, 0.35
Venezuela	**0.88, 0.50, 0.64**	0.57, 0.31, 0.40	0.56, 0.26, 0.36	0.65, 0.12, 0.20
Ecuador	**0.82**, 0.51, 0.63	0.72, 0.44, 0.55	0.54, **0.93**, **0.68**	0.62, 0.71, 0.66

#### Effectiveness of LMSS

In this set of experiments, we evaluated the effect of utilizing LMSS as a component of our method by comparing its performance against that of the baseline method DQE + SS described above. The results of the comparison are shown in Table 6. DQE + LMSS clearly outperforms DQE + SS, achieving much higher F-measures in most of the countries tested except Brazil and Argentina. DQE + SS has F-measure values below 0.5 in half of the countries, and its recall values are lower than 0.3 in 3 countries. The superior performance demonstrated by both DQE and LMSS vindicate the decision to utilize them as the components of the proposed event detection method.

### Event Detection Performance

Our proposed approach was compared with existing methods based on precision, recall, and F-measures on civil unrest event detection.

The experimental results are illustrated in Table 7, which shows that the proposed method achieves the best overall performance. Except for Brazil, Ecuador, and Paraguay, DQE + LMSS achieves the highest F-measures in every country. Even for these 3 countries, it scored the best on precision and achieved a highly competitive overall performance. Although TEDAS also achieves a relatively good performance compared to the other benchmark methods, it still produced 4 countries with F-measures lower than 0.5. Among the existing methods, the Earthquake method and EDSS were relatively advantageous in precision, but suffered from a limited ability to detect most of the events. ST Burst performed better in large countries such as Brazil, Argentina, and Mexico, than in the smaller ones. Graph Partition and Topic Modeling, which are unsupervised methods designed for events under general-interest domain, seem relatively weak for detecting events under a targeted domain, achieving F-measures over 0.5 in very few countries.

**Table 7:** Performance Comparison with Existing Event Detection Methods (Precision, Recall, F-measure).

Dataset	DQE + LMSS	Graph Partition	Earthquake	Topic Modeling	TEDAS	ST Burst	EDSS
Brazil	**0.93**, 0.37, 0.53	0.55, 0.34, 0.42	0.65, 0.19, 0.30	0.46, 0.09, 0.15	0.39, 0.20, 0.27	0.80, **0.45**, **0.58**	0.86, 0.28, 0.42
Colombia	0.81, **0.75, 0.78**	0.68, 0.29, 0.41	0.55, 0.49, 0.52	0.26, 0.39, 0.31	0.66, 0.41, 0.50	**0.87**, 0.48, 0.62	0.57, 0.52, 0.54
Uruguay	0.66, **0.82, 0.73**	0.28, 0.23, 0.25	0.86, 0.11, 0.20	0.22, 0.06, 0.09	**0.88**, 0.56, 0.68	0.11, 0.06, 0.08	0.66, 0.13, 0.22
El Salvador	**0.83, 0.43, 0.56**	0.35, 0.07, 0.10	0.32, 0.06, 0.10	0.40, 0.05, 0.09	0.71, 0.36, 0.48	0.30, 0.12, 0.17	0.52, 0.15, 0.23
Mexico	**0.91, 0.49, 0.64**	0.72, 0.23, 0.35	0.51, 0.19, 0.28	0.34, 0.08, 0.12	0.56, 0.20, 0.29	0.76, 0.43, 0.55	0.69, 0.27, 0.39
Chile	0.80, **0.69, 0.74**	0.83, 0.39, 0.53	0.46, 0.19, 0.27	0.42, 0.48, 0.45	**0.96**, 0.36, 0.53	0.67, **0.69**, 0.68	0.35, 0.43, 0.39
Paraguay	**0.98**, 0.35, 0.52	0.76, 0.19, 0.30	0.40, 0.10, 0.16	0.86, 0.07, 0.13	0.88, **0.67, 0.76**	0.34, 0.12, 0.18	0.83, 0.16, 0.27
Argentina	0.78, 0.61, **0.69**	**0.88**, 0.14, 0.24	0.63, 0.57, 0.60	0.38, 0.42, 0.40	0.51, **0.64**, 0.57	0.63, 0.73, 0.67	0.73, 0.55, 0.63
Venezuela	**0.88, 0.50, 0.64**	0.46, 0.21, 0.29	0.87, 0.22, 0.35	0.47, 0.37, 0.41	0.79, 0.28, 0.42	0.82, 0.33, 0.47	0.86, **0.50**, 0.63
Ecuador	**0.82**, 0.51, 0.63	0.30, 0.22, 0.25	0.78, **0.60**, 0.68	0.67, 0.04, 0.08	0.55, 0.92, **0.69**	0.29, 0.26, 0.27	0.64, 0.28, 0.39

The computation times consumed by these methods are shown in Table 4. There is no significant difference in running times among most of the methods. The only exception is Topic Modeling, which took less than 10 hours. Note that unlike the other targeted-domain spatial event detection methods, namely Earthquake and TEDAS, our method is unsupervised, which means it does not need to devote additional effort to labeling.

In summary, the experiments clearly demonstrate the effectiveness and efficiency of the proposed DQE + LMSS approach.

### Study of Parameters

The impact of the parameters of the proposed approach was evaluated, including (i) *N*, the number of the seed query terms, (ii) *β*, the parameter for updating tweet node weights (see Equation 2), (iii) 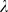, the trade-off between local modularity and spatial scan statistics (see Equation 7), and (iv) *r*, the longest distance between any two neighbor nodes.

**Figure 3:**
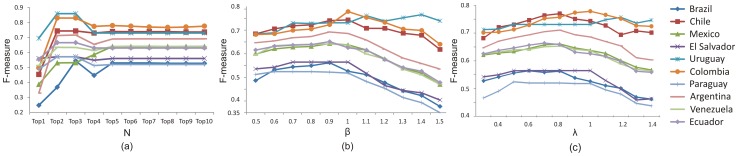
Sensitivity analysis of parameters. (a) Sensitivity analysis of “number of seed query terms” (b) Sensitivity analysis of “trade-off *β* for updating tweet node weights” (c) Sensitivity analysis of “trade-off 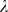 between local modularity and spatial scan statistics”.

Figure 3(a) illustrates the performance of our method versus *N*, the number of seed query terms. For most of the countries, the F-measures corresponding to *N* = 2 or 3 are significantly higher than when *N* = 1. But when *N* increases further, the F-measures tend to be stable, especially once *N* reaches 5.

Figure 3(b) shows the results of varying *β* from 0.5 to 1.5. By increasing the value of *β* to around 1, the F-measures of most countries are improved, but once it exceeds 1, the performance drops.

The results of tuning 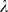 are shown in Figure 3(c). By varying 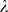 from 0.3 to 1.4, the F-measures generally increase, reaching their peaks when 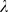 is in the range of 0.7 to 1.2. This suggests the “sweet region” of 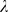 to correspond to the point where the local modularity and spatial scan statistics combine to achieve the optimal performance. Moreover, even with an extreme value of 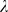, say 0.3 or 1.4, the overall performance of the proposed model remains highly competitive compared to its peers, as can be seen in the data shown in Table 7.

**Figure 4:**
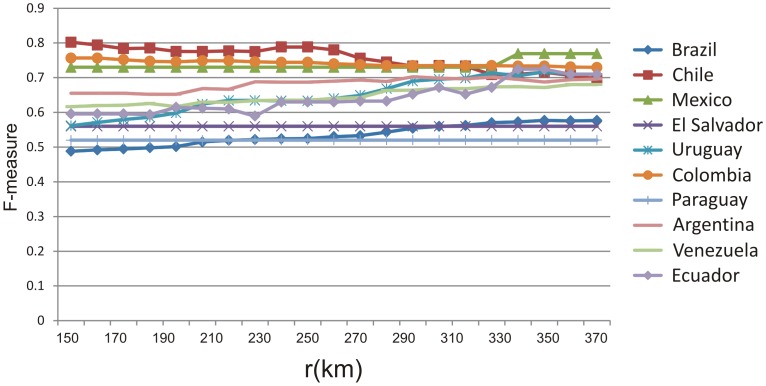
Sensitivity analysis of the longest distance *r* between any two neighboring locations.

Figure 4 illustrates the F-measures obtained by varying *r* from 150 km to 370 km. The F-measures for Colombia, Paraguay, Mexico, El Salvador, and Argentina do not change significantly with respect to *r*. For the other 5 countries, the effect on the F-measures of varying the value of *r* are mostly less than 0.1, which are still minimal.

### Case Study

**Figure 5:**
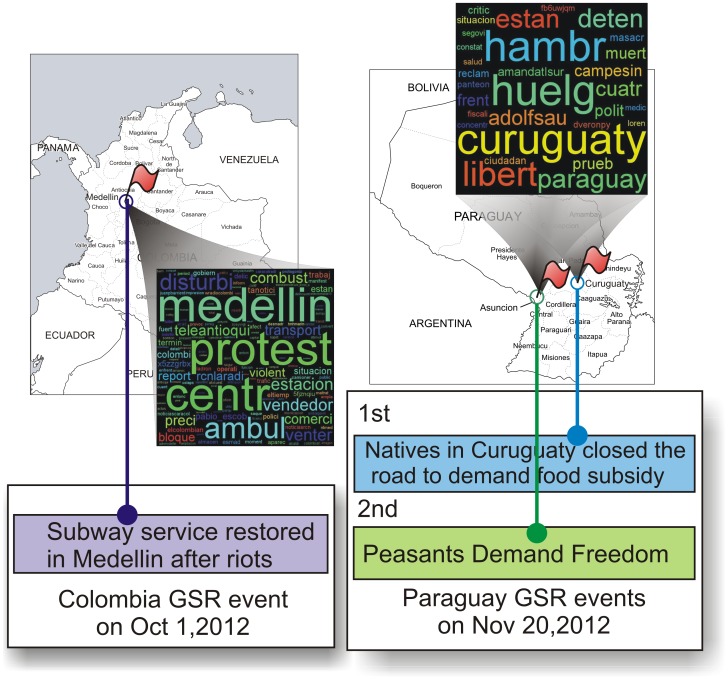
Event detection case studies.

During the experiments, a number of interesting facts revealed by using the proposed approach was observed. For instance, comparing the results for Colombia and Paraguay, the very different expanded query terms from these two countries reflect their correspondingly different social foci, which contributed to the model's ability to accurately detect local events accordingly. As shown in Figure 5, the major term for movements is “protest” in Colombia (as on October 1, 2012) versus “huelga” (i.e., “strike” in English) in Paraguay (as on November 20, 2012). The cities “Medellin” in Colombia and “Curuguaty” in Paraguay were both hot spots for unrest events, but the movements in Colombia seem more metropolitan-related, because of the appearance of terms such as “estacion” (station), “transport”, and “teleantioqui” (television). Paraguay's themes for these events are more about “libert”, “campesin” (peasant), and “hambr” (hunger). These cases reveal that our method can indeed capture the variety of keywords across different countries. It is worth noting that ongoing unrest keywords, even in the same country, tend to evolve over time, as shown in Table 5, and our DQE can still capture this evolution effectively.

Based on the expanded queries generated by our DQE, LMSS was able to identify spatial unrest events. In the above examples, as shown in Figure 5, the proposed method detected one event on October 1st, 2012 in Colombia that was related to transportation in the city of Medellin; on Nov. 20th, 2012 in Paraguay, the proposed method detected 2 events concerned with about “food subsidy” in Curuguaty and “peasants demand freedom” in Asunción, respectively.

## Conclusion

This paper presents a novel unsupervised approach for detecting spatial events under targeted domains. We developed dynamic query expansion that utilizes a Twitter heterogenous information network to dynamically extract domain-related key terms. To extract spatial events based on these domain-related tweets, we designed a local modularity spatial scan capable of simultaneously considering the semantic similarity and the geographical proximities of tweets. Extensive empirical studies on civil unrest event detection were conducted based on Twitter data collected in 10 Latin American countries. The results demonstrated the effectiveness and efficiency of our proposed approach.
